# Advancing Survival in Nigeria: A Pre-post Evaluation of an Integrated Maternal and Neonatal Health Program

**DOI:** 10.1007/s10995-018-2476-3

**Published:** 2018-02-09

**Authors:** Nancy L. Sloan, Andrew Storey, Olufunke Fasawe, Jamila Yakubu, Kelly McCrystal, Owens Wiwa, Lene Jeanette Lothe, Mari Grepstad

**Affiliations:** 1 New York, USA; 2 Clinton Health Access Initiative, 383 Dorcester Avenue, Suite 400, Boston, MA 02127 USA; 3 Clinton Health Access Initiative, 7B Ganges St. Maitama, Abuja, Nigeria; 4 Norwegian Agency for Development Cooperation, Bygdøy Allé 2, 0257 Oslo, Norway

**Keywords:** Maternal mortality ratio, Neonatal mortality rate, Stillbirth rate, Perinatal mortality rate, Nigeria

## Abstract

*Introduction* Nigeria contributes more obstetric, postpartum and neonatal deaths and stillbirths globally than any other country. The Clinton Health Access Initiative in partnership with the Nigerian Federal Ministry of Health and the state Governments of Kano, Katsina, and Kaduna implemented an integrated Maternal and Neonatal Health program from July 2014. Up to 90% women deliver at home in Northern Nigeria, where maternal mortality ratio and neonatal mortality rates (MMR and NMR) are high and severe challenges to improving survival exist. *Methods* Community-based leaders (“key informants”) reported monthly vital events. Pre-post comparisons of later (months 16–18) with conservative baseline (months 7–9) rates were used to assess change in MMR, NMR, perinatal mortality (PMR) and stillbirth. Two-tailed cross-tabulations and unadjusted and adjusted logistic regression analyses were conducted. *Results* Data on 147,455 births (144,641 livebirths and 4275 stillbirths) were analyzed. At endline (months 16–18), MMR declined 37% (OR 0.629, 95% CI 0.490–0.806, p ≤ 0.0003) vs. baseline 440/100,000 births (months 7–9). NMR declined 43% (OR 0.574, 95% CI 0.503–0.655, p < 0.0001 vs. baseline 15.2/1000 livebirths. Stillbirth rates declined 15% (OR 0.850, 95% CI 0.768–0.941, p = 0.0018) vs. baseline 21.1/1000 births. PMR declined 27% (OR 0.733, 95% CI 0.676–0.795, p < 0.0001) vs. baseline 36.0/1000 births. Adjusted results were similar. *Discussion* The findings are similar to the Cochrane Review effects of community-based interventions and indicate large survival improvements compared to much slower global and flat national trends. Key informant data have limitations, however, their limitations would have little effect on the results magnitude or significance.

## Significance

Most obstetric and neonatal mortality continues to occur where access to care is limited. In July 2014, an integrated Maternal and Neonatal Health program was implemented in Northern Nigeria, where ~90% women deliver at home, to provide immediate quality community-based lifesaving services and increased interaction with the formal health system.

Compared to baseline, there were large, highly significant increases in women’s 37%, neonatal 43% and perinatal 27% survival and a 15% stillbirth reduction. The findings are biologically plausible and consistent with the recent Cochrane Review effects of community-based interventions and indicate large survival improvements compared to global and national trends.

## Introduction

Nigeria contributes the largest number of global obstetric and postpartum deaths (58,000, 19.1% of 303,000) (World Health Organization 2015; World Health Organization 2016), neonatal deaths (261,549, 12.5% of 2,100,000) (World Health Organization 2016; Liu et al. 2012) and stillbirths (301,267, 11.6% of 2,600,000) of any country (Blencowe et al. 2016). In lower and middle income countries (LMIC), hemorrhage, sepsis and eclampsia account for half of all women’s deaths and over 80% of neonatal deaths and stillbirths are caused by complications of preterm birth, intrapartum events, and infections (Liu et al. 2012). In Nigeria, the national maternal mortality ratio (MMR) and neonatal mortality rate (NMR) are 576/100,000 and 37/1,000 livebirths, respectively (National Population Commission (NPC) [Nigeria] and ICF International 2014), with a stillbirth rate of 43/1000 births (Blencowe et al. 2016). The highest rates occur in the impoverished Northwest region, where severe challenges to improving health care and survival exist, including poor access and transportation to reach health care, and where up to 90% of women deliver at home (National Population Commission (NPC) [Nigeria] and ICF International 2014).

## Intervention

**Fig. 1:**
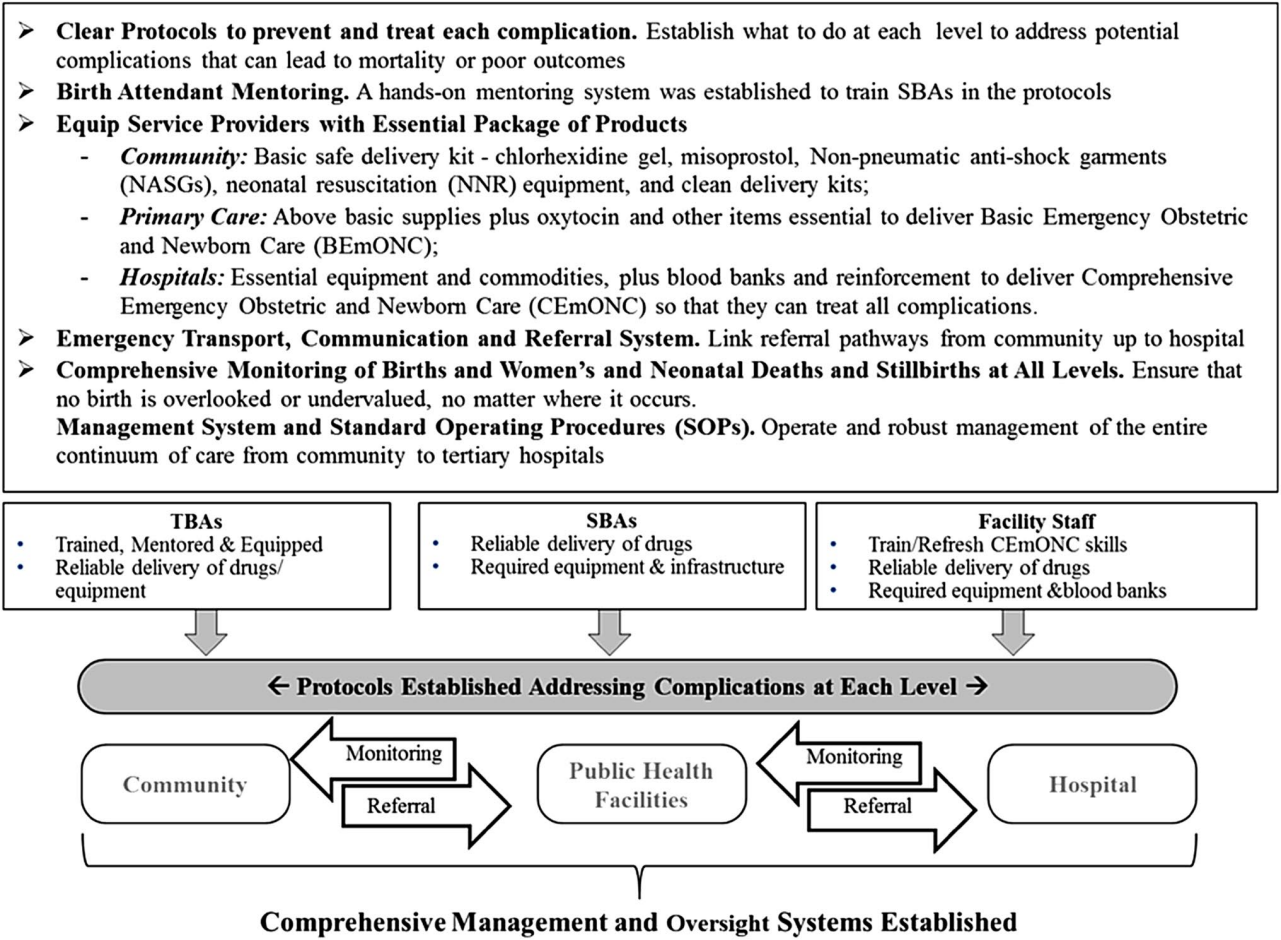
The three state MNH integrated approach to ensure a continuum of care from the community through to the hospital level

**Fig. 2:**
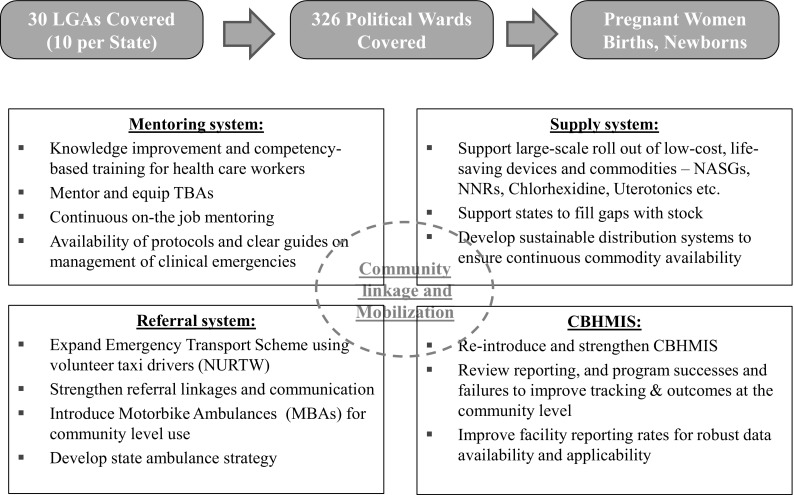
Implementation of the integrated three state MNH integrated approach establishing and reinforcing linkages across levels of care

In response to the challenges, the Clinton Health Access Initiative (CHAI) reviewed previous efforts in Nigeria and concluded that a holistic, integrated approach was needed to efficiently use scarce donor and government resources to improve women’s and neonatal health outcomes. In July 2014, the Government of Norway provided financial support to the Nigerian Federal Ministry of Health for CHAI to work in partnership with the state Governments of Kano, Katsina, and Kaduna to develop and implement an integrated Maternal and Neonatal Health (MNH) program. Kano, Katsina, and Kaduna have among the highest MMR and NMR in the country, accounting for approximately 20% of women’s and neonatal deaths in Nigeria as estimated from Demographic Health Survey (DHS) data (National Population Commission (NPC) [Nigeria] and ICF International 2014). Consistent with World Health Organization (WHO) guidelines (World Health Organization 2013), including averting preventable deaths occurring within 48 h of birth, the approach addresses critical gaps in care by linking the health system, from household to hospital. Because traditional birth attendants (TBAs) had little education and training, TBAs had only been allowed to refer complications. As most complications and death occur in the community, TBAs and facility-based Skilled Birth Attendants (SBAs) were trained, equipped and mentored to be effective first responders. TBAs 35 to 60 years old, with at least 8 years experience and reasonable vision known to facility in-charges from previous community engagement activities were selected. The 2791 selected TBAs were formally incorporated into the health care system and received a 4 days (3 days didactic, 1 day hands-on practice) training session in January 2015. The TBA training covered early identification of women’s and neonatal danger signs, oral misoprostol for postpartum hemorrhage (PPH) prevention with, clearing neonate’s nose and mouth, chlorhexidine for cord care, and first-aid management of complications, including identifying PPH as postpartum bleeding which soaked two wrappers (sarongs) and application of Non-Pneumatic Anti-Shock Garments (NASG) for PPH, and manual neonatal resuscitator (NNR) to stabilize patients and timely emergency referral and transport to the nearest health care facility or hospital. State certified senior nurses and midwives conducted the training. All TBAs were linked to their nearest health care facility whose staff mentored them for 6 months. One focal TBA was selected with whom eight to ten TBAs from the same geographic ward would meet with on a weekly basis to practice their skills, discuss and resolve issues and to replenish their supplies. Between January and March, 2015, certified community health extension workers (CHEWs), midwives and nurses at primary care facilities providing antenatal care and/or labor and delivery in the program Local Government Authorities (LGAs), were trained in Life Saving Skills (LSS) and Essential Newborn Care (ENC), and equipped to deliver Basic Emergency Obstetric and Newborn Care (BEmONC). To ensure skills breadth and transfer in the case of staff turnover, all the antenatal and labor and delivery care staff received 2 weeks training (6 days didactic, 6 days hands-on practice) conducted by government certified master trainers, mostly doctors, including obstetricians/gynecologists. In March 2015, doctors and senior nurses from secondary level facilities were trained on NASG application and in neonatal resuscitation with mannequins, and were equipped to deliver Comprehensive Emergency Obstetric and Newborn Care (CEmONC). The program distributed 3800 NASGs and 3000 NNRs to TBAs and health facilities. In May 2015, a 3 months mentoring program was implemented for the primary and secondary level providers. In all, 1645 nurses, midwives and CHEWs were trained and 1450 were mentored. To ensure timely and prompt referral to the right level of care, a functional voluntary emergency transport and communications system was established with the National Union of Road Transport Workers (NURTW). Two hundred and fifty motorbike ambulances (MBAs) were provided in June-July, 2015with 1338 drivers identified and trained by October, 2015. Community leaders usually retained the MBAs to facilitate community member access, TBAs and community members were trained to call the MBA or ambulance driver who would call the nearest facility to ensure readiness for patient reception or to be re-directed to another prepared facility. If re-directed, the facility focal person would call the higher level facility to prepare themselves to receive the emergency patient. Aggregate program data indicate ~ 34,000 referrals and ~ 20,000 transfers were made. Figures 1 and 2 summarize the project interventions. The program approach was designed to provide immediate quality community-based lifesaving services and increased interaction with the formal health system, creating a continuum of care, fomenting trust and demand while facilities become more prepared to provide obstetric, postpartum and neonatal care. The program is ongoing in 30 (of 101) of the highest mortality LGAs without other known systematic MNH programs in the three focal states, targeting a population of approximately ten million.

## Objective

The objective of this assessment was to evaluate the MNH program impact on reducing women’s, neonatal and perinatal mortality and stillbirth.

## Methods

### Study Design

A pre-post design was used to evaluate change in mortality by comparing later with baseline incidence rates or ratios. In November, 2014, the MNH program revitalized, upgraded and expanded a dormant Community Based Health Management Information System (CBHMIS) in which key informants (KIs) reported monthly births, women’s and neonatal deaths and stillbirths. The KIs were mostly respected traditional community leaders with basic primary or higher education. In the case of death or disability, the new community leader became the KI. All traditional leaders had a scribe with basic primary or higher education who could read and write English or Hausa. As with the interventions, the CBHMIS was established to promote local ownership.

### Procedures

A monitoring and evaluation (M&E) officer for each state, and various Local Engagement Consultants (LECs), who were communities/LGA members, mostly with previous similar data collection experience and CBHMIS familiarity, were engaged and trained by CHAI staff to oversee data collection. In November, 2014, the M&E officers led their state’s initial 2 days LGA level training sessions of the district heads, KIs and their scribes on the use of the program tools, forms, data templates and on the reporting structure. The training included information on MMR, NMR, SBR and their major causes and means of prevention. As a result of a CHAI site data audit in October 2015 that identified data irregularities and under-reporting, LGA level review meetings were held monthly through July 2015 and quarterly thereafter. Each M&E officer created a report of trends and gaps shared by the LECs at the meetings to resolve data quality issues. LECs also reviewed survival trends to identify and resolve program implementation limitations and share successes achieved to continue motivated and accurate data collection. The meetings produced a dramatic rise in reported events and stable reporting from May 2015 onwards. The M&E officers provided ongoing supervision to the LECs on a daily to weekly basis, depending on the geographic span of the LEC coverage area.

From November 2014, over 1,500 KIs reported vital events for settlements (groups of 5–10 households) in the program LGAs. Data were collected with pen on paper, recording a ‘1’ as a stillbirth, livebirth or neonatal death and the women’s age (years) at delivery or at neonatal death (days) in Kano and Katsina. Kaduna reported gender only for deaths. Because gender influences survival (Sawyer 2012), the team retroactively classified missing gender using neonates’ (livebirths who survived or died ≤ 28 days) first names. Facility-based births and deaths were reported by families to the KIs. To maintain data collection simplicity, multiple gestation (which increases mortality risk) and individual exposure to specific interventions were not recorded, thus their effects could not be directly assessed. State was systematically reported. Data were collected at the lowest(settlement)level and reported up to the traditional ward, then the village, the District LGA (where data entry in Excel occurred), then to the LEC, state M&E officer, and finally to the CHAI national M&E officer where the data files were aggregated. Hard copy forms for out-of-range or inconsistent data reported between January 2015 and June 2016 were reviewed to correct electronic records. The Excel spreadsheets were securely transferred and imported into SPSS for Windows version 23 for analysis.

### Outcomes

The primary outcomes were women’s and neonatal mortality and stillbirth; perinatal mortality was a secondary outcome (see definitions in Table 1).

**Table 1:** Evaluation outcomes

	Definition
Primary outcomes	
Maternal mortality ratio (MMR)	# Women’s deaths (age 14–45)/100,000 all births
Neonatal mortality rate (NMR)	# Neonatal deaths (births ≥ 28 weeks estimated by last menstrual period or, if unavailable, women’s self-reported gestation with any sign of life or that cried before dying < 29 days)/1000 livebirths
Stillbirth rate	# Stillbirths (births ≥ 28 weeks estimated by last menstrual period or, if unavailable, women’s self-reported gestation without signs of life (not breathing or no heartbeat at birth) even after attempted resuscitation)/1000 all births
Secondary outcomes	
Perinatal mortality rate (PMR)	# Stillbirths + # early neonatal deaths (age < 8 days)/1000 all births

### Statistical Analysis

The evaluation (report to be posted on http://www.clintonhealthaccess.org along with an independent qualitative evaluation conducted by KPMG) analyzed CBHMIS vital events data and reviewed information from CHAI staff and program documents. The KIs were becoming familiar with data reporting between January and May 2015. Half as many births were reported in quarter 1 (Q1) and two-thirds in Q2 compared with Q3–Q5 (Table 2). Further training and monitoring stabilized the number of births reported from June 2015 onwards. As under-estimation of births would spuriously inflate mortality rates and over-estimate program effect, the evaluation conservatively used quarter 3 data (July–September 2015), when all the interventions were fully rolled out, as the baseline comparison group. Quarter 6 was the endline. More births were reported in Q6, with an increase in reporting villages and their population, but the similarity of Q5 and Q6 results (described below) suggests little (e.g., parallel if any) bias.

**Table 2:** Numbers of Births (Live and Stillbirths) and LGAs by State and Quarter

State	Quarters						Total	LGAs
	Q1 1–3/15	Q2 4–6/15	Q3 7–9/15	Q4 10–12/15	Q5 1–3/16	Q6 4–6/16		
Kano	5702	9073	12,930	12,498	13,351	19,291	72,845	10 (of 44)
Katsina	3448	6616	10,976	11,632	12,943	14,671	60,286	10 (of 34)
Kaduna	5542	7686	9027	8123	9358	12,655	52,391	10 (of 23)
Total	14,692	23,375	32,933	32,253	35,652	46,617	185,522	30 (of 101)

Cross-tabulations with Chi square statistics and unadjusted and adjusted logistic regression analyses were conducted for all outcomes. Where inconsistent or out-of-range data correction was not possible, missing values were assigned for analysis. The regression Odds Ratios (OR), the 95% confidence intervals (CI), and two-tailed significance values (p) are presented. Neonatal mortality regression models were adjusted for gender and to adjust geographic differences in socio-economic status and program implementation state. All remaining outcomes were adjusted for state only. For adjusted analyses, missing gender was re-coded to the mean value. Data were analyzed in quarterly periods to minimize variability in event reporting by settlement. All 30 program LGAs contributed to each quarter. Variability in the number of monthly reporting settlements might influence the effects, however data were not adjusted for clustering given the large number of settlements and the small number of quarterly births and deaths per settlement, which nearly represents individual-level reporting. In the absence of extreme reporting bias, with the very large numbers reported upon, and large and highly significant results, adjustment for such clustering would have little effect on the findings magnitude or significance.

**Fig. 3:**
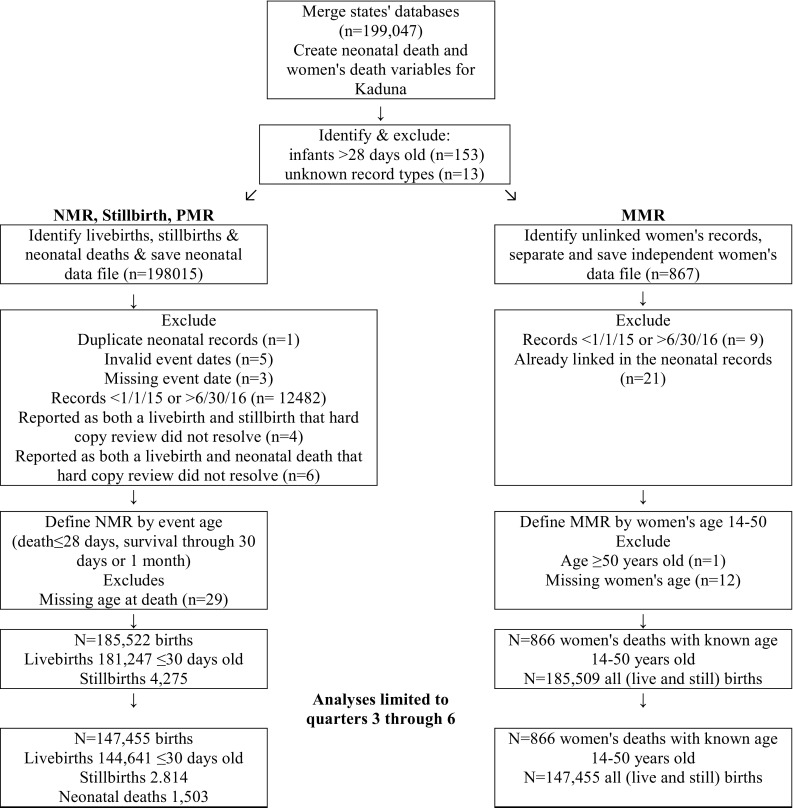
Flow diagram and process used to merge and analyze event registry files

As the vital events system reported all deaths, MMR and NMR analyses assume survival through 42 days postpartum and 28 days of life, respectively. Assuming neonates and women who did not die survived through 28 and 42 days, respectively, and excluding the few infant deaths beyond 28 days or women’s deaths from antepartum hemorrhage that did not result in births may minimally under-represent the denominators but with very large numbers of births, should have little effect on the results magnitude or significance. The three women aged 14 and the five aged 45–50 years were included in analysis as plausible obstetric and postpartum deaths (Fig. 3). No deliveries or deaths were reported in girls less than 14 years old. Only one women’s death > 50 years was reported; she was 60 years and excluded from analysis. Women (n = 12) and neonates (n = 29) with missing age were excluded from analysis. The timing of women’s deaths was not reported.

Women’s and infants’ events were almost all reported as separate records, without unique linking identifiers. To attain the most accurate MMR denominator, the best woman-infant links were accomplished using geographic, event date and name fields. With imperfect matching, all (still and live) births constitute the MMR denominator; all women’s deaths are the numerator.

## Ethics

The program and its data collection system were approved by both the federal and program states’ governments. The program evaluation is considered exempt under 45 CFR 46.101(b) from all 45 CFR and does not require IRB approval according to the Office of Human Research Protection (OHRP) guidance on exemptions at http://www.hhs.gov/ohrp/policy/index.html#exempt.

## Findings

The CBHMIS reported more annual vital events than most and even multi-country household surveillance systems (Kirkwood et al. 2010; Goudar et al. 2012). Data on 185,522 births (181,247 livebirths and 4275 stillbirths) from Q1 to Q6 were analyzed. As women’s deaths with unknown age (n = 12) or age > 50 (n = 1) were excluded, 185,509 records (including livebirths and stillbirths) were analyzed for MMR (Fig. 3). The analyses of quarters 3 through 6 include 147,455 births (144,641 livebirths and 2814 stillbirths).

**Fig. 4:**
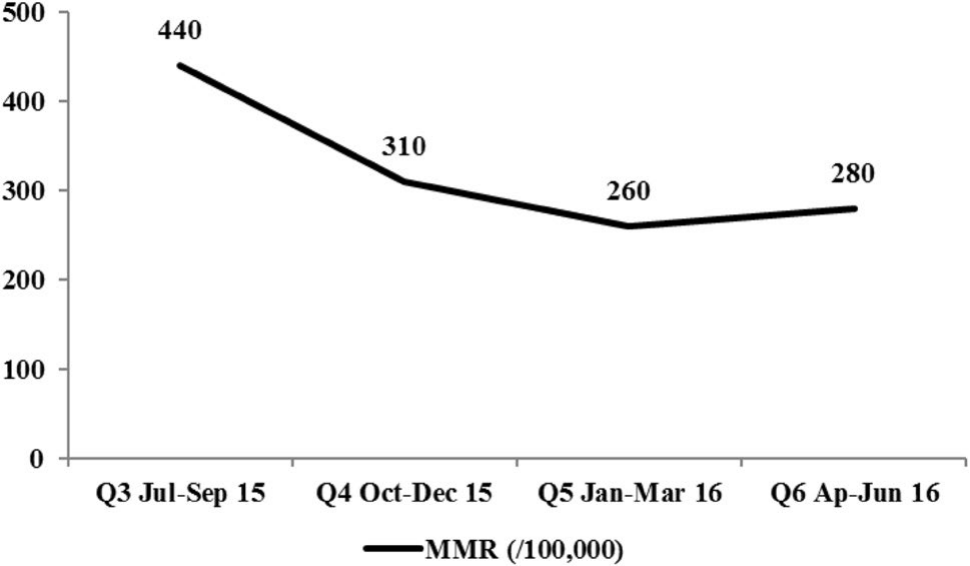
Maternal mortality ratios per 100,000 births by quarter

Between Q3 (July–September 2015) and Q6 (April–June 2016) 1291 women’s deaths were reported. Women’s mortality declined across all three states (Table 3; Fig. 4). Kano and Kaduna Q6 MMR had significantly lower MMR (p = 0.0074 and p = 0.0001, respectively) and Katsina’s Q6 MMR was substantially lower (p = 0.0641, marginally significant) than their Q3 baselines. A highly significant 37% decline in Q6 compared with the Q3 MMR of 440/100,000 births was observed, (OR 0.629, 95% CI 0.490–0.806, p ≤ 0.0003, Table 4), with a 28% Q4 decline (OR 0.718, 95% CI 0.551–0.937, p = 0.0147) and a 39% Q5 decline (OR 0.629, 95% CI 0.490–0.806, p ≤ 0.0003). Adjustment for state had little effect.

**Table 3:** MMR, NMR, Stillbirth (SB) and PMR by State and quarter (Q); and comparison of outcomes for Q3 vs Q6

MMR^a^	Q3		Q4		Q5		Q6		Q6 vs. Q3comparison
State	N	MMR	N	MMR	N	MMR	N	MMR	
Kano	12,929	340	12,495	376	13,350	157	19,289	249	0.0074
Katsina	10,975	565	11,632	318	12,943	371	14,669	416	0.0641
Kaduna	9027	443	8123	209	9358	246	12,655	158	0.0001
Total	32,931	443	32,250	313	35,651	258	46,613	277	0.0003

NMR^b^	Q3		Q4		Q5		Q6		Q6 vs. Q3comparison
State	N	NMR	N	NMR	N	NMR	N	NMR	
Kano	12,662	14.6	12,238	8.6	13,104	8.7	18,933	8.0	< 0.0001
Katsina	10,723	17.4	11,363	10.4	12,690	10.6	14,374	10.8	< 0.0001
Kaduna	8854	13.3	7989	8.6	9239	7.7	12,472	7.6	< 0.0001
Total	32,239	15.2	31,590	9.2	35,033	9.1	45,779	8.8	< 0.0001

Stillbirth^c^	Q3		Q4		Q5		Q6		Q6 vs. Q3comparison
State	N	SB	N	SB	N	SB	N	SB	
Kano	12,930	20.7	12,498	20.8	13,351	18.5	19,291	18.6	0.2934
Katsina	10,976	23.1	11,632	23.1	12,943	19.5	14,671	20.2	0.1046
Kaduna	9027	19.2	8123	16.5	9358	12.7	12,655	14.5	0.0028
Total	32,933	21.1	32,253	20.6	35,652	17.4	46,617	18.0	0.0002

PMR^c^	Q3		Q4		Q5		Q6		Q6 vs. Q3comparison
State	N	PMR	N	PMR	N	PMR	N	PMR	
Kano	12,930	35.0	12,498	29.2	13,351	27.0	19,291	26.4	< 0.0001
Katsina	10,976	40.1	11,632	33.3	12,943	29.9	14,671	30.8	< 0.0001
Kaduna	9027	32.2	8123	25.0	9358	20.3	12,655	22.0	< 0.0001
Total	32,933	36.0	32,253	29.6	35,652	26.3	46,617	26.6	< 0.0001

^a^Per 100,000 births

^b^Per 1,000 livebirths

^c^Per 1,000 births

**Table 4:** Percent distribution of Neonatal Gender by State

State	Kano		Katsina		Kaduna		Total	
	Male	Female	Male	Female	Male	Female	Male	Female
N	38,133	34,702	29,418	30,863	18,099	34,041	85,650	99,606
%	52.4	47.6	48.8	51.2	34.7	65.3	46.2	53.8

Newborns with missing gender were excluded from analysis

Over all quarters (Q1–Q6) between January 2015 and June 2016, 185,522 births were reported, 72,845 in Kano, 60,286 in Katsina and 52,391 in Kaduna (Table 2); 46.2% were classified as male and 53.8% female (Table 5). The male-to-female birth ratio in Kano was 1.10:1 and 0.95:1.00 in Katsina, but was 0.53:1.00 in Kaduna and likely reflect misclassification where most neonatal gender was assigned retroactively based on the infants’ first names.

**Table 5:** Unadjusted and Adjusted Logistic Regressions of MMR, NMR, Stillbirth, PMR Comparing Quarters 4, 5 and 6 with Q3

	OR	95% Lower CI	95% Upper CI	p	OR	95% Lower CI	95% Upper CI	p
MMR (N = 147,455)	Unadjusted				Adjusted			
Q 3 (baseline)	Reference group				Reference group			
Q 4	0.718	0.551	0.937	0.0147	0.709	0.543	0.925	0.0112
Q 5	0.606	0.462	0.794	0.0003	0.597	0.455	0.783	0.0002
Q 6	0.629	0.490	0.806	0.0003	0.634	0.494	0.812	0.0003
Katsina^a^	–	–	–	–	1.492	1.203	1.850	0.0003
Kaduna^a^	–	–	–	–	0.916	0.706	1.188	0.5068

NMR (N = 144,641)	Unadjusted				Adjusted			
Q 3 (baseline)	Reference group				Reference group			
Q 4	0.605	0.523	0.699	< 0.0001	0.601	0.519	0.695	< 0.0001
Q 5	0.595	0.517	0.686	< 0.0001	0.592	0.513	0.682	< 0.0001
Q 6	0.574	0.503	0.655	< 0.0001	0.576	0.505	0.658	< 0.0001
Gender^b^	–	–	–	–	0.786	0.709	0.871	< 0.0001
Katsina^a^	–	–	–	–	1.253	1.115	1.408	0.0001
Kaduna^a^	–	–	–	–	0.974	0.851	1.115	0.7011

Stillbirth (N = 147,455)	Unadjusted				Adjusted			
Q 3 (baseline)	Reference group				Reference group			
Q 4	0.975	0.876	1.086	0.6440	0.968	0.869	1.078	0.5509
Q 5	0.821	0.736	0.916	0.0004	0.816	0.732	0.911	0.0003
Q 6	0.850	0.768	0.941	0.0018	0.851	0.769	0.942	0.0019
Katsina^a^	–	–	–	–	1.096	1.007	1.192	0.0340
Kaduna^a^	–	–	–	–	0.794	0.719	0.877	< 0.0001

PMR (N = 147,455)	Unadjusted				Adjusted			
Q 3 (baseline)	Reference group				Reference group			
Q 4	0.818	0.750	0.892	< 0.0001	0.812	0.745	0.886	< 0.0001
Q 5	0.725	0.664	0.790	< 0.0001	0.720	0.660	0.786	< 0.0001
Q 6	0.733	0.676	0.795	< 0.0001	0.734	0.677	0.796	< 0.0001
Katsina^a^	–	–	–	–	1.146	1.07	1.227	< 0.0001
Kaduna^a^	–	–	–	–	0.839	0.774	0.909	< 0.0001

^a^Kano is the reference state

^b^Male is the reference gender

Of the 183, 473 livebirths reported between Q1 and Q6, the average and standard deviation, and the median age at death in days for neonates were 6.41 ± 6.29 and 5.00 (with an interquartile range [IQR] 8.00) days (n = 1503). Neonatal age at death was fairly stable over time (data not shown), although fewer died in the first 2 days of life in Q5 and Q6. Thirty-six percent of neonates’ deaths were age ≤ 1 day in Q3, 42% in Q4, 32% in Q5 and 30% in Q6. Age at death was 1 day greater in Q5 and Q6 than Q3 and Q4.

**Fig. 5:**
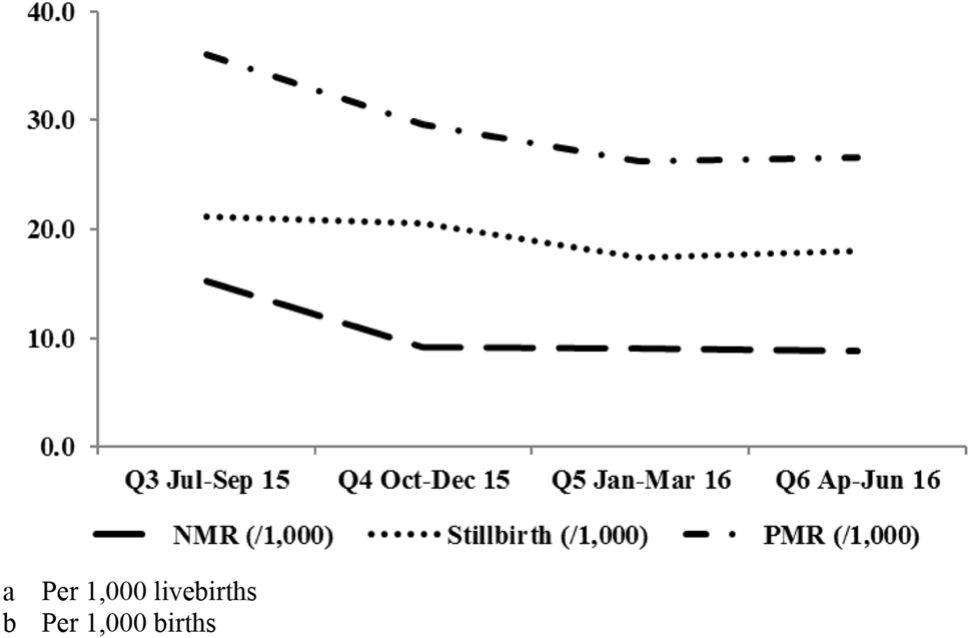
Neonatal mortality (Per 1000 livebirths), stillbirth (Per 1000 births) and perinatal (Per 1000 births) mortality rates by quarter

NMR declined sharply (p ≤ 0.001) between Q3 (July–September 2015) and Q6 (April–June 2016; Table 3; Fig. 5). Compared with the Q3 baseline rate of 15.2/1,000 livebirths, a 43% decline in at Q6 was observed (OR 0.574, 95% CI 0.503–0.655, p < 0.0001, Table 4), with declines of 39% and 40% in Q4 (OR 0.605, 95% CI 0.523–0.699, p < 0.0001) and Q5 (OR 0.595, 95% CI 0.517–0.686, p < 0.0001). Adjustment for gender and state had little effect.

Stillbirth rates declined between Q3 and Q6 (Table 3; Fig. 5), but were only statistically significant at the state level in Kaduna. A 15% decline in stillbirth (OR 0.850, 95% CI 0.768–0.941, p = 0.0018) was observed between Q3 and Q6 and an 18% decline in Q5 (OR 0.821, 95% CI 0.736–0.916, p ≤ 0.0004), with little change between Q3 and Q4 (OR 0.975, 95% CI 0.876–1.086, p = 0.6440), Table 4. Adjustment for state had little effect. Progress to reduce LMIC stillbirths has been slow (Sather et al. 2010; Little et al. 2010; Goldenberg et al. 2016). Most neonatal mortality occurs within the first few days of life (Sankar et al. 2016), when NMR occurs disproportionately in very ill neonates. A stillbirth to NMR ratio of 0.90:1.00 is expected from high quality data, with a ratio of 0.75:100 in poor quality data (Blencowe et al. 2016). Mis-reporting neonatal deaths on the day of birth as stillbirths is a common problem worldwide (Blencowe et al. 2016; Lawn et al. 2010). The observed ratio of stillbirth to neonatal mortality was about 2.00:1.00, indicating that many neonatal deaths were mis-reported as stillbirths and explains why the program had smaller stillbirth than NMR and PMR benefit.

PMR declined (p < 0.0001) between July 2015 and June 2016 (Table 3; Fig. 5). A 27% PMR decline (OR 0.733, 95% CI 0.676–0.795, p < 0.0001) was observed between Q3 and Q6, and a 23% Q5 decline (OR 0.725, 95% CI 0.664–0.790, p < 0.0001, Table 4). As PMR reflects stillbirth and early NMR, an intermediate 18% PMR change was observed between Q3 and Q4 (OR 0.818, 95% CI 0.750–0.892, p < 0.0001). Adjustment for state had little influence. The baseline PMR is almost identical (36/1000) to the most recent DHS for the north-central Nigeria (34/1000), supporting the validity of the program PMR estimates (National Population Commission (NPC) [Nigeria] and ICF International 2014).

## Discussion

Efforts to improve survival in Nigeria have had poor to mixed results. The three state Nigeria MNH program findings suggest that the comprehensive, integrated program accomplished substantial, sustained and highly significant reductions in women’s mortality (37%), neonatal mortality (43%), stillbirth (15%) and perinatal mortality (27%).

Much of the observed program effect may be attributable to the implementation of simple, highly effective interventions rooted in community-based training and involvement. Community-based neonatal stimulation without bag and mask ventilation reduced stillbirth by 24% in rural India (Goudar et al. 2013). Sound rural LMIC studies found oral misoprostol reduced postpartum hemorrhage by 36% and NASG reduced PPH case-fatality by over 50% (Sloan et al. 2010; Miller and Belizán 2015). In rural northern India, a comprehensive community-based ENC package reduced NMR by 54%, attributing much of the effect to skin-to-skin care, immediate breastfeeding and first postnatal bathing ≥ 24 h after delivery (Kumar et al. 2008). Community-based application of chlorhexidine for umbilical cord care has been shown to reduce NMR by 19% (Sinha et al. 2015).

The observed effects are quite similar to the results reported by the most recent Cochrane Review of community-based interventions, supporting their consistency and biologic plausibility (Lassi and Bhutta 2015). That review found community-based interventions in various countries increased the use of simple, effective interventions, particularly early initiation of breastfeeding, TBA/SBA clean delivery kit use and emergency transfers, as did the integrated program approach. The Cochrane Review found community mobilization and ante- and post-natal visitation and home-based treatment packages also conducted under challenging circumstances generally reduced NMR by 37–40% with 95% CIs from a 20% increase to a 70% NMR decrease. The review found similar interventions reduced MMR by an average 26–28% with 95% CIs covering a 20% increase to a 50% MMR decrease. Over all community-based interventions, the review found a 20% stillbirth reduction covering a 10% increase to a 40% decrease, and a 22% reduction in PMR covering a 20% increase to a 45% decrease. When limited to well-controlled studies with low risk of bias, the average reductions were 30% NMR, 24% MMR, 25% stillbirth and 27% PMR. In contrast, a recently published large multi-country randomized controlled trial of a similar integrated approach to improve BEmONC and CEmONC, including SBA and TBA training, community mobilization and enhanced referral not yet included in the Cochrane reviews had little or no effect on MMR, NMR, stillbirth or PMR (Pasha et al. 2013). Even in that trial, diverse conditions and implementation across sites may account for the large NMR and PMR reductions observed in rural India that were not observed elsewhere (Goudar et al. 2015).

To avoid over-estimation of effect and to reflect when all the interventions were fully rolled out, using Q3 as the baseline may under-estimate the program effect as most interventions were initiated in January 2015 before Q3. The most recent DHS reports an NMR of 44/1000 livebirths in Northwest Nigeria between 2004 and 2013 and a national rate of 37/1000 livebirths in 2013 (National Population Commission (NPC) [Nigeria] and ICF International 2014). Even if Q1 and Q2 births were under-reported, their NMRs of 22.6/1000 and 18.0/1000 livebirths were substantially lower, indicating that the program had some additional earlier effect. In contrast, the DHS maternal mortality ratio (e.g., the ratio of reproductive age women’s deaths to livebirths) between 2006 and 2013 was 576 with a 95% CI of 500–652 per 100,000 livebirths. Although the evaluation’s MMR denominator includes both livebirths and stillbirths, the Q1 and Q2 MMRs were considerably higher, 1310/100,000 and 880/100,000 births (live and still). Consistent with the evaluation’s larger MMR denominator (including stillbirths), the Q3 evaluation MMR estimate of 440/100,000 was below the lower DHS MMR 2006–2013 CI, and the MMR was similar to the lower CI of the 2001–2008 DHS estimates of 475/100,000 livebirths (National Population Commission (NPC) [Nigeria] and ICF International 2014). Unlike NMR, however, DHS samples do not provide accurate point and interval estimates for MMR. Using reports on deaths of women of reproductive age, however, contributes to the strength of the evaluation as a funnel for identifying suspect obstetric and postpartum deaths. With the very large sample, the 95% confidence intervals indicate reliable and, except for stillbirth, strong effect boundaries.

Key informant data collection has limitations and may not ensure data quality equivalent to professional prospective surveillance or periodic household surveys (Silva et al. 2016; Silva 2012; Joos et al. 2016). Similar to the program’s KI system, facility-based integrated programs using quality improvement systems, to provide timely information for program decisions in Niger and Mali increased attention to monitor and manage all pregnancies and thus contributed to improving survival (Boucar et al. 2014). Professionally conducted household surveillance was initially considered but sufficient funding was unavailable. Future professional assessment with household mapping that efficiently expands the breadth of data (Christian et al. 2013; D’souza 1981) to evaluate receipt and use of the interventions is recommended.

The evaluation had no contemporaneous control group to help attribute survival improvement to the program (Miller et al. 2003), and to avoid unexpected potential harm. To avoid contamination, LGAs where other organizations were conducting MNH programs were excluded from the MNH program. Temporal change unassociated with the program could decrease or increase the observed effects. Nigeria has experienced stable or increased neonatal and women’s mortality over the past years (National Population Commission (NPC) [Nigeria] and ICF International 2014), whereas the program area experienced substantial and significant mortality reductions. In the absence of extreme bias or activities unknown to the program staff, the effects’ magnitude and significance is likely attributable to the program. UNICEF estimated that NMR reduced by 47% between 1990 and 2015 (UNICEF Global databases 2016) and WHO and the United Nations Maternal Mortality Estimation Inter-Agency Group estimates MMR reduced by 44% in that same time period (World Health Organization 2015; Alkema et al. 2016). The three state MNH Program in Nigeria observed and sustained similar results in 12 months between July 2015 and June 2016. The capacity building of government staff instituted at the beginning of the program supported a gradual transition of program responsibilities. The interventions’ effects peaked in Q4, 9 months after the program was initiated. CBHMIS data not available at the time of this evaluation indicates continued survival improvement after a period of stabilization between Q5 and Q6, when program responsibilities were successfully transitioned to the government.

In conclusion, compared to much slower global and flat national trends, the evaluation results indicate large survival improvements are most likely attributable to the MNH program and should be scaled up. In a resource constrained setting with high obstetric, postpartum, perinatal and newborn mortality, and previously disjointed MNH care, a coherent, integrated approach simultaneously addressing community, primary health care and CEmONC care can greatly improve women’s and neonatal survival.
